# *Doenitin-1*: A novel Kunitz family protein with versatile functions during feeding and reproduction of the tick *Haemaphysalis doenitzi*

**DOI:** 10.3389/fvets.2022.872244

**Published:** 2022-08-10

**Authors:** Jialin Lu, Kuang Wang, Zhihua Gao, Songbo Zhang, Hao Li, Yanqing Shi, Xuecheng Song, Jingze Liu, Zhijun Yu, Xiaolong Yang

**Affiliations:** Hebei Key Laboratory of Animal Physiology, Biochemistry and Molecular Biology, College of Life Sciences, Hebei Normal University, Shijiazhuang, China

**Keywords:** *Haemaphysalis doenitzi*, thrombin inhibitor, anticoagulant activity, blood-sucking, hemolysis activity

## Abstract

As obligate blood-feeding ectoparasites, ticks secrete a great diversity of antithrombin molecules during feeding. In this study, a novel antithrombin gene named *Doenitin-1* was characterized from the tick *Haemaphysalis doenitzi*. It has an open reading frame size of 426 bp; it encodes 141 amino acids and has a predicted molecular weight of 15.8 kDa. The fibrinogen coagulation test showed that the time of coagulation was increased significantly with increase in rDoenitin-1 protein concentration, and the activated partial thromboplastin time (APTT) and prothrombin time (PT) assays showed that rDoenitin-1 significantly prolonged the coagulation time of APTT, indicating that rDoenitin-1 has an anticoagulant activity *in vitro*. In addition, rDoenitin-1 presents a significant inhibitory activity in thrombin and cathepsin G. The hemolysis rate of rDoenitin-1 in healthy human blood cells was 4.25%, and no obvious hemolysis activity was observed. The comparison with other life stages shows that the higher expression occurs in adults, and tissue comparison indicated a higher expression in the midgut. The RNAi results indicated that interference of *Doenitin-1* significantly reduced the engorgement rate and egg hatchability of *H. doenitzi*, and that the engorged body weight was slightly reduced. In conclusion, the results suggested that the novel gene *Doenitin-1* functions in blood-feeding of *H. doenitzi* and performs various functions during feeding and reproduction of *H. doenitzi*. *Doenitin-1* may be a potential vaccine candidate for tick control and for developing new antithrombotic drugs in the future.

## Introduction

Ticks are obligate blood-feeding ectoparasites that can transmit a wide diversity of pathogens, including bacteria, viruses, and protozoans, to their hosts, causing great damages to wild animals, affecting livestock production, and threatening human health (1). Ixodid ticks usually feed for several days or weeks on their hosts and secrete many functional molecules during feeding (2). Among them, anticoagulation molecules play important roles in facilitating tick feeding (3). Anticoagulation is mainly attributed to inhibition of the clotting cascade, especially the key coagulation factors FXa and thrombin (4). Thrombin is usually composed of 36–259 amino acids; it can hydrolyze specific peptide bonds in fibrinogen to form insoluble fibrin and activate platelets and clotting factors to enhance clotting cascade (5). Therefore, a thrombin inhibitor is the most common anticoagulant molecule found in blood-sucking parasites to ensure successful blood-feeding and survival (6, 7). To date, several anticoagulants have been identified from the midgut, salivary glands, and hemolymph of ticks (Table 1). These molecules have a direct effect by keeping the blood in liquid state to ensure successful feeding of ticks (23).

**Table 1:** Thrombin inhibitors in ticks.

**Genus**	**Species**	**Anticoagulant molecules**	**Conserved domains**	**Clotting mechanism**	**References**
*Haemaphysalis*	*H. longicornis*	Madanin 1	Thrombin inhibitor Madanin	Inhibit thrombin	(8)
		Madanin 2	Thrombin inhibitor Madanin	Inhibit thrombin	(8)
		Hemalin	BPTI/Kunitz	Inhibit thrombin	(9)
	*H. bispinosa*	Haemathrins 1&2	Thrombin inhibitor Madanin	Inhibit thrombin	(10)
*Ixodes*	*I. ricinus*	Ixin		Inhibit thrombin	(11)
	*I. scapularis*	Ixophilin	BPTI/Kunitz	Inhibit thrombin	(12)
*Amblyomma*	*A. americanum*	Variegin		Inhibit thrombin	(13)
	*A. variegatum*	Americanin		Inhibit thrombin	(14)
*Argas*	*A. monolakensis*	Monobin	BPTI/Kunitz	Inhibit thrombin	(15)
*Ornithodoros*	*O. moubata*	Ornithodorin		Inhibit thrombin	(16)
	*O. savignyi*	Savignin		Inhibit thrombin	(17)
*Hyalomma*	*H. dromedarii*	NTI-1		Inhibit thrombin&FXa	(18)
	*H. dromedarii*	NTI-2		Inhibit thrombin&FXa	(18)
	*H. marginatum*	Hyalomin-1		Inhibit thrombin	(8)
	*H. dromedarii*	Dromaserpin	Serine protease inhibitor-associated domains s3a and RCL	Inhibit thrombin, Kallikrein, FXIa and slightly FXIIa	(19)
*Rhipicephalus*	*R. microplus*	BmAP	A1 family peptidase	Inhibit thrombin	(20)
		Microphilin		Inhibit thrombin	(20)
		Boophilin	BPTI/Kunitz	Inhibit thrombin	(21)
		RmS-15	serpin family	Inhibit thrombin	(22)

ND, None.

Thrombin inhibitors are major members of the serine peptidase inhibitor family and have some biological activities like other family members. For example, the boophilin from *Rhipicephalus* (*Boophilus*) *microplus* was found to inhibit trypsin, neutrophil elastase, and bovine thrombin (21). Similarly, the anticoagulant molecules hemathrins 1 and 2 showed inhibitory effects on serine peptidases, such as thrombin, trypsin, and plasmin (10). However, the peptidases with Kunitz domains found in ticks may not only have an antithrombin function, but they also regulate host immunity (24, 25).

*Haemaphysalis doenitzi* is a three-host tick and widely distributed in South China, including in Fujian, Hainan, Yunnan, and Taiwan (26). In this study, a novel antithrombin gene was characterized from the tick *H. doenitzi*, and its inhibition of serine peptidase activity and hemolytic activity was assayed. Subsequently, potential functions were explored during blood-feeding and reproduction of *H. doenitzi* in the hope of identifying potential vaccine candidates for tick control and future development of new antithrombotic drugs.

## Materials and methods

### Collection and feeding of ticks

Wild adult *H. doenitzi* ticks were collected from vegetation by flag-dragging in Cangxi county (31°37′-32°10′N, 105°43′-106°28′E), Sichuan province. They were placed on the ears of New Zealand white rabbits glued with cloth bags for feeding. Those in non-parasitic stages are kept in an incubator at 26 ± 1°C and 75 ± 3% relative humidity under a 16/8-ligh/dark photoperiod. All experiments in this study were administrated with the permission of the Animal Ethics Committee of the Hebei Normal University (Protocol Number: IACUC-156027).

### RNA extraction, cDNA synthesis, cloning, and sequence analysis

A number of unfed adult ticks with total body weights ranging from 50 to 80 mg were randomly selected and extracted for RNA according to the instructions of the TransZol™ Up Plus RNA Kit (TransGen Biotech, China). A cDNA library was synthesized according to Easy Script^®^ first-strand cDNA Synthesis SuperMix (TransGen Biotech, China) and stored at −80°C.

Based on the transcriptome library of *H. doenitzi* deposited on GenBank (accession number: MZ981733), specific primers (forward primer 5′-CAGCGAAATGGCTTCTG-3′ and reverse primer 5′-TCAGTATTTGCAGGCGAAC-3′) were designed using Primer Premier 5.0 (Premier Biosoft International, United States). The PCR program was as follows: 94°C for 10 min, followed by 28 cycles each at 94°C for 30 s, 56°C for 30 s, 72°C for 1 min, and then a final elongation at 72°C for 10 min. The target PCR product was isolated by agarose gel electrophoresis (1%) and purified with gel extraction kits. Subsequently, the purified PCR product was inserted into the pMD^®^19-T Simple Vector (BGI, China) for sequencing. The confirmed sequence was analyzed by BLAST on NCBI (http://blast.ncbi.nlm.nih.gov/Blast.cgi). Multiple sequence alignments were performed using the DNAMAN software (http://www.lynnon.com). The analysis selected *H. longicornis* (BAH02683.1)*, H. flava* (ANA67892.1)*, R. microplus* (XP_037274758.1)*, D. variabilis* (ACF35510.1), *and I. scapularis* (XP_029835135.2). Then, the molecular weight (MW) and theoretical isoelectric point (pI) were predicted using Compute PI/Mw (http://web.expasy.org/compute_pi/). Signal peptides of the sequence were predicted based on SignalP (http://www.cbs.dtu.dk/services/SignalP/).

### Spatiotemporal expression patterns of *Doenitin-1*

A total of 120 partially fed female adult ticks were used to collect hemolymph, salivary glands, ovaries and midguts, and eggs (incubated for 10 days); larvae (10 days after hatching), nymphs (10 days after molting), and adults (10 days after molting) were used to determine different life stages (unfed) and different tissue expression patterns of *Doenitin-1*. Total RNA was extracted as described above, and specific primers (qRT-PCR forward primer 5′-ATGGCTTCTGTCGGCTTCC-3′ and qRT-PCR reverse primer 5′-TTAGAGTCTCGAAGTTGTTCTCGTT-3′) were used. β*-actin* was used as a reference gene (β*-actin* forward primer 5′-CGTTCCTGGGTATGGAATCG-3′ and β*-actin* reverse primer 5′-TCCACGTCGCACTTCATG-3′) (27). Then, the SPSS 16.0 software for Windows (SPSS Inc, USA) was used to conduct a one-way ANOVA for qRT-PCR results (28).

### Expression and purification of *Doenitin-1* recombinant protein

The fragment was inserted into pET-32a with the *Not I* and *BamH I* restriction sites and then transformed into *Escherichia coli* (TransGen Biotech, China) cells and cultured overnight at 37°C. We added IPTG until the final concentration was 0.2 mM, and *E. coli* was induced for 6 h at 200 r/min at 37°C. The bacteria were centrifuged at 12,000 × g for 10 min, and the resultant supernatant was filtered with a 0.4-μm filter.

Recombinant proteins were purified using a ProteinIso™ Ni-NTA Resin kit (TransGen Biotech, China). The sample was added to the chromatography column filled with Ni-NTA for affinity for 30 min. Then, it was eluted with different imidazole concentrations (20, 50 100, 200, 250, and 500 mM) to get the recombinant protein in the gravity affinity chromatography. Finally, after elution with a 3-ml elution buffer (0.025 M NaCl, 0.05 M NaHPO_4_, and 0.02 M imidazole, pH = 7.4), the chromatography column was sealed with 3 ml anhydrous ethanol.

Preliminary purified samples were lyophilized and then diluted to a suitable concentration with ddH_2_O. Subsequently, dialysis membranes with a molecular weight cutoff of 8–14 kDa (Solarbio, China) and an ultrafiltration centrifugal tube (3 kDa) were used to further purify the eluted recombinant protein. Aliquots of a 20-μl sample (1 mg/ml) was boiled for 5 min and then separated by SDS-PAGE (12%). The concentration of the purified protein was determined using a BCA protein detection kit (Transgenic, China). The purified recombinant protein was identified by LC-MS/MS using a M-Class nanoACQUITY ultra performance liquid chromatography (UPLC) (Waters, USA) and a Q Exactive HF mass spectrometer (Thermo Fisher Scientific).

### Activated partial thromboplastin time (APTT) and prothrombin time (PT) assays

Healthy adult human venous blood (2 ml) was collected in anticoagulant tubes containing sodium (KWS, China) and centrifuged at 4,000 r/min for 5 min to separate the plasma. The activated partial thromboplastin time (APTT) kit and the prothrombin time (PT) kit were purchased from Sunbio (China). The plasma and reagents were incubated at 37°C for 3 min. First, we used an ACLTOP 700 coagulation analyzer (Werfen, Spain) to test APTT and PT as the group of control. The results were recorded, and three normal samples were selected (PT normal value: 12–16 s; normal APTT: 24–36 s). Second, the recombinant protein was diluted at different concentrations of 0, 3.5, 7, 14, and 28 μM. Then, the groups were added separately to normal plasma samples, and the APTT and PT results were recorded after re-mixing. The experiment was repeated thrice.

### Fibrinogen coagulation test

After mixing 80 μl fibrinogen solution and 80 μl rDoenitin-1 with different concentrations (0, 0.5, 1, 1.5, 2, and 2.5 μM), the mixtures were mixed and placed at a 37°C constant temperature water bath and incubated for 2 min. Then, 80 μl thrombin (Sigma, China) was added, and the absorbance of the mixture was immediately determined at 650 nm every 12 s with a UV spectrophotometer and monitored for 30 min. The experiment was repeated thrice.

### Inhibitory effect of rDoenitin-1 on serine peptidase activity

rDoenitin-1 was mixed with three serine peptidases (thrombin, trypsin, and cathepsin G; Sigma, China). According to Assumpção et al. (29), corresponding chromogenic substrates were added to detect the residual activity of the peptidase. The buffers selected for the reaction of thrombin, trypsin, and cathepsin G with recombinant proteins were 50 mM Tris-HCl (pH 8), 150 mM NaCl, 20 mM CaCl_2_, and 0.01% Triton X-100. Boc-ASP-Pro-Arg-AMC (24.2 μM), Boc-GLN-Ala-Arg-AMC (250 μM), and SUC-Ala-Ala-Pro-Phe-AMC (250 μM) (Njpeptide, China) were selected as the chromogenic substrate for thrombin (27 nM), trypsin (10 nM) and cathepsin G (10 nM), respectively, and the concentration was 24.2 μM when the substrate was dissolved in a buffer under dark conditions. Dissolved thrombin at a concentration of 27 nM was used. The recombinant protein was diluted at a concentration gradient (32, 16, 8, 4, 2, 1, and 0.5 nM for thrombin; 200, 100, 50, 25, 12.5, and 6.25 nM for trypsin and cathepsin G) and no recombinant protein at 0 nM. Serine peptidase (20 μl) and recombinant protein (20 μl) with different concentration gradients were placed into an incubator and incubated at 37°C for 30 min (40-μl buffer was added into the control well). On a 96-well black ELISA plate (Corning, United States), a 60-μl buffer was added to each well, followed by the addition of a 100-μl color developing substrate under dark conditions. The microplate was then put into a microplate meter; the excitation wavelength was set at 355 nm and the emission wavelength at 460 nm to measure the OD value of each well. The experimental group OD value was A_sample_, the recombinant protein concentration at 0 nM was A_black_, and the control well OD value was A_control_. Residual peptidase activity (%) = (A_sample_-A_control_)/A_black_ × 100% (30). The experiment was repeated thrice.

### Hemolytic activity test

The healthy adult venous blood (6 ml) was mixed with 6 ml normal saline and centrifuged for 4 min at 3,000 × g, and the supernatant discarded. An appropriate amount of normal saline was added and centrifuged for 4 min at 3, 000 × g, and the supernatant was discarded. We repeated the previous step until the supernatant of the liquid changes from red to transparent after centrifugation. We added 9 ml normal saline to suspend the packed red blood cells and added the following solution into a 1.5-ml EP tube according to the following system: positive control group (absorbance value is A): 20 μl 1%Triton X-100 and hematocyte suspension 980 μl; negative control group (absorbance value is B): 20 μl normal saline and hematocyte suspension 980 μl; experimental group (absorbance value is C): 20 μl recombinant protein and hematocyte suspension 980 μl. The reagents were placed in a 37°C constant temperature water bath and incubated for 1 h; 300 μl of the supernatant was collected after centrifugation at 3,000 × g for 3 min. The absorbance value of each group was determined with a UV spectrophotometer at 540 nm. Three replicates were performed for each group.

Three repeated tests were performed to calculate the hemolysis rate of rDoenitin-1 according to the following formula: hemolysis rate =[(C–B)/(A–B)] × 100%.

### Functions of *Doenitin-1* during feeding and reproduction of *H. doenitzi*

The versatile functions of *Doenitin-1* during feeding and reproduction of *H. doenitzi* were explored using RNAi. Briefly, the primers including the T7 promoter sequence at the 5′-end of both primers (*Doenitin-1* primer: 5′-TAATACGACTCACTATAGGAACTGGCTGCAAGCCAG-3′ and 5′- TCAGTATTTGCAGGCGAAC-3′; 5′-AACTGGCTGCAAGCCAG-3′ and 5′- TAATACGACTCACTATAGGTCAGTATTTGCAGGCGAAC-3′; GFP primer: 5′-TAATACGACTCACTATAGGGACGTAAACGGCCACAAGT-3′ and 5′- GCTTCTCGTTGGGGTCTTT-3′; 5′-GACGTAAACGGCCACAAGT-3′ and 5′- TAATACGACTCACTATAGGGCTTCTCGTTGGGGTCTTT-3′) were used to synthesize dsRNA. Unfed female and male ticks with uniform body size were selected, and the interference group was injected with *Doenitin-1* dsRNA, while the control group was injected with GFP dsRNA. About 0.6 μl dsRNA (2,400 ng) was injected into the fourth basal segment of the abdomen of each female tick under a microscope using a beveled Hamilton's needle (10 μl). The injected ticks were placed in an incubator and observed for 24 h. After 24 h of observation, dead or weak ticks were removed, and ticks in the interference group and the control group were put into ear bags with a ratio of 20 female ticks to 15 male ticks per rabbit ear. Twenty-four hours later, the number of feeding ticks was checked to calculate the rate of attachment rate. After 4 days, 2–3 partially fed female ticks were removed from each group to determine the relative expression of the genes by qPCR. The experimental data were analyzed according to the 2^−ΔΔCt^ algorithm, and the RQ value of each sample was calculated according to the formula and analyzed by biostatistics with the SPSS software. The time, body weight, and engorgement rate were recorded after the ticks were collected. Engorged female ticks were placed in an incubator, and the oviposition rate and hatching rate of eggs were recorded.

### Statistical analysis

A statistical analysis was performed using SPSS 13.0 (SPSS Inc., Chicago, IL). Data organization and analysis were performed with Graphpad Prism 8.0.1. The statistical method were one-way ANOVA and Student's *t* test. Whether there is a significant difference between different samples is indicated by different letters. All the data were expressed as mean ± standard deviation (SD) or percentage. *P* < 0.05 was considered statistically significant.

## Results

### Sequence analysis and spatiotemporal expression

**Figure 1:**
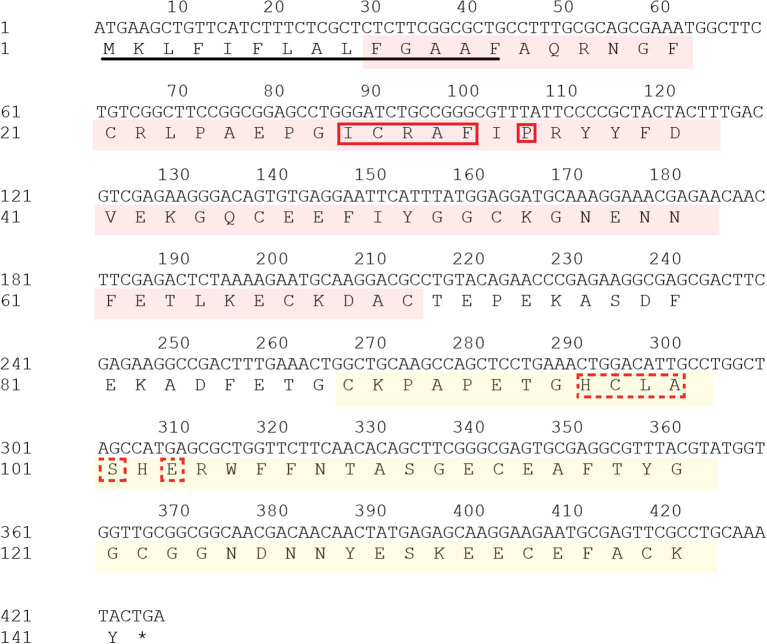
Nucleotide sequence and deduced amino acid sequence of *Doenitin-1*. Row 1: Number position, which is counted every 10 bases. Row 2: Nucleotide sequence. Row 3: Amino acid sequence. The red area is the BPTI/Kunitz family of serine protease inhibitor domain; the yellow area is the Kunitz/Bovine pancreatic trypsin inhibitor domain; signal peptides are underlined; the solid red box is the BPTI/Kunitz binding presenting loop; the red dotted box is the Kunitz/Bovine pancreatic trypsin binding presenting loop. Both domains belong to the BPTI/Kunitz family of serine protease inhibitors.

**Figure 2:**
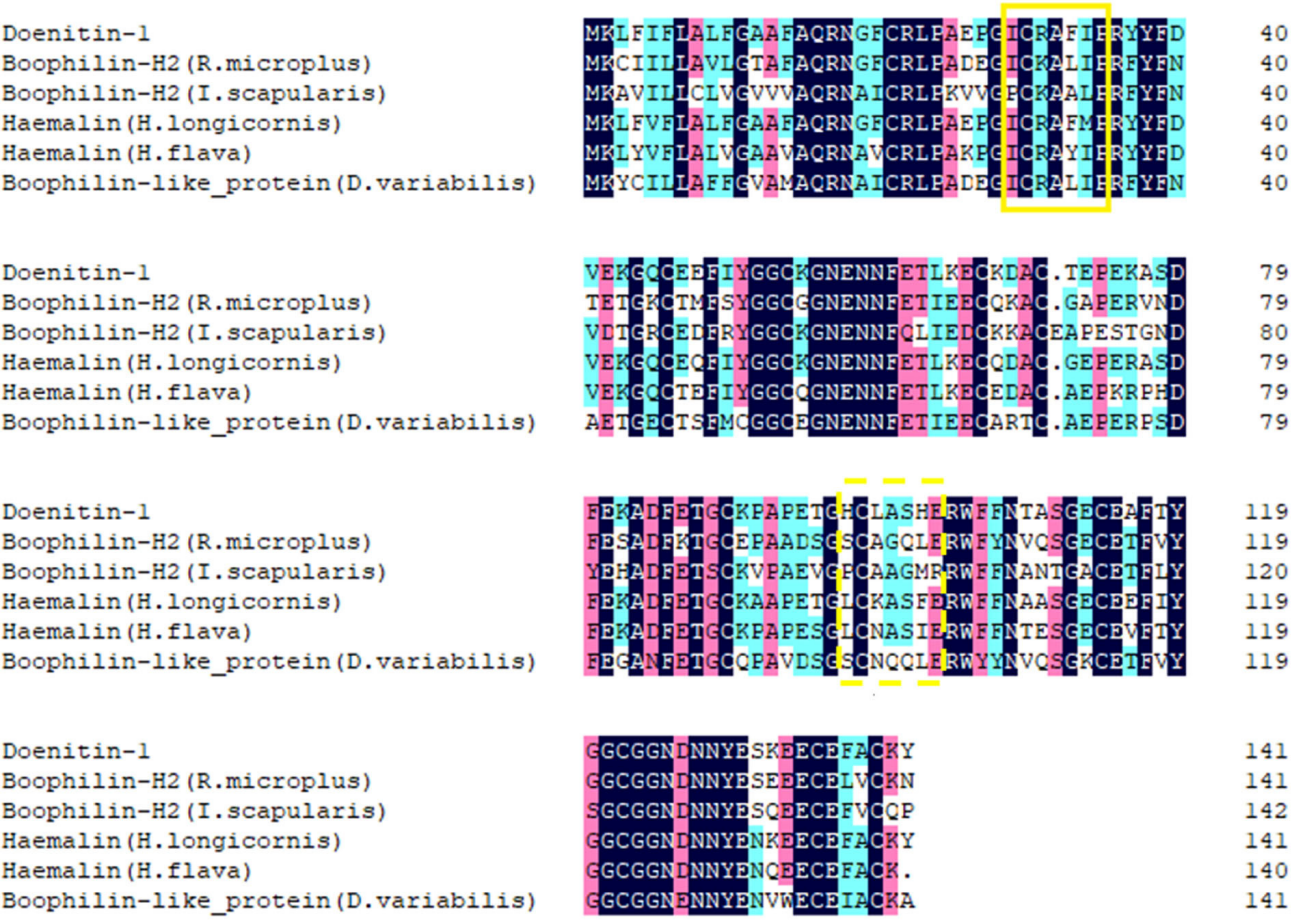
Alignment of *Doenitin-1* with its homologs in other tick species. The identical amino acid sequence is in the last row. The solid yellow box is the BPTI/Kunitz substrate binding presenting loop; the yellow dotted box is the Kunitz/Bovine pancreatic trypsin binding presenting loop. The black highlight homology level equals 100%, the pink highlight homology level is greater than or equal to 75%, the blue is greater than or equal to 50%.

The open reading frame (ORF) of *Doenitin-1* (accession number: MZ981733) is 426 bp and encodes 141 amino acids (Figure 1). It is predicted that *Doenitin-1* is 15.8 kDa, and that the theoretical isoelectric point (pI) is 4.76. It is presumed that *Doenitin-1* contains a signal peptide of 15 amino acids and two Kunitz domains. Identity-based BLAST searches showed that 90% identity was found with *H. longicornis* (BAH02683.1), 83% identity with *H. flava* (ANA67892.1), 66% identity with *R. microplus* (Q8WPI2.1), 63% identity with *Dermacentor variabilis* (ACF35510.1), and 59% identity with *Ixodes scapularis* (XP_002434145.1). The multiple sequence alignment is shown in Figure 2.

**Figure 3:**
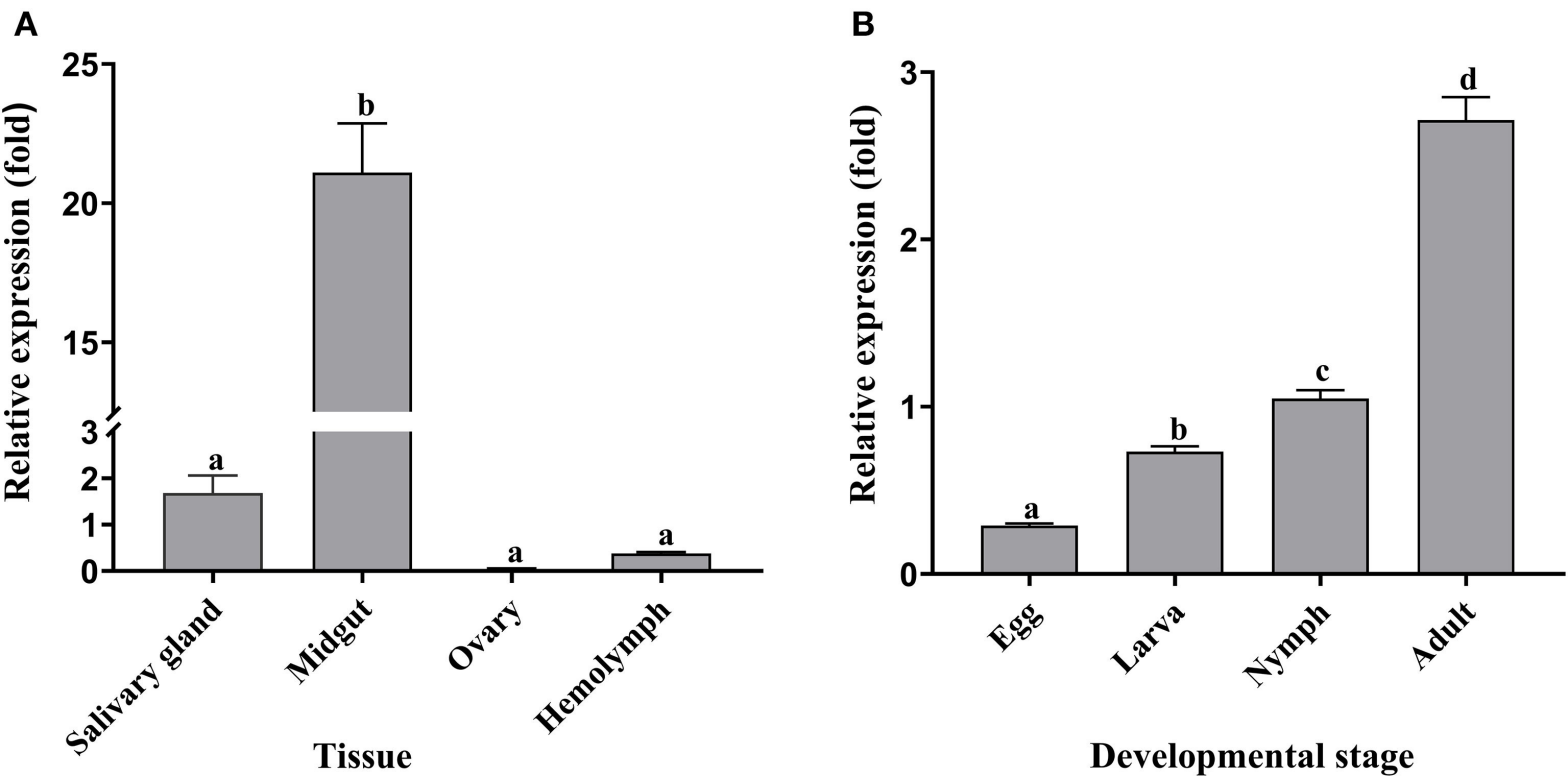
Spatiotemporal expression level of *Doenitin-1* in *H. doenitzi*. **(A)** Different tissues of *Doenitin-1*. **(B)** Different stages of *Doenitin-1*. The SPSS 16.0 software for Windows (SPSS Inc, IL, United States) was used to conduct a one-way ANOVA for qRT-PCR results. Different letters indicate statistical differences (*P* < 0.05).

The qRT-PCR results showed that *Doenitin-1* was expressed in all tissues (partially fed) and stages (unfed) (Figure 3). By one-way ANOVA, the expression level of *Doenitin-1* in the midgut was significantly higher than in other tissues (*P* < 0.05) (Figure 3A), and the highest expression level was found in unfed adult ticks (Figure 3B).

### Test of APTT and PT of rDoenitin-1

**Figure 4:**
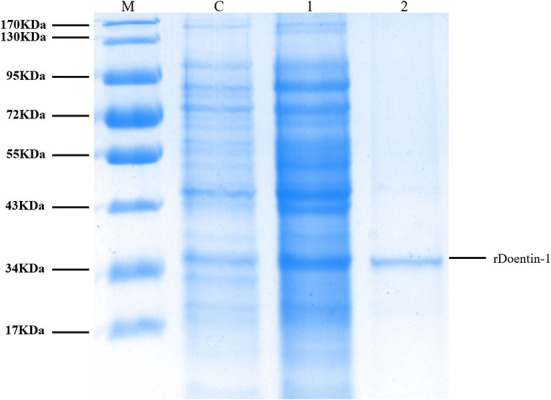
SDS-PAGE (12%) gel profile of rDoenitin-1 stained with Coomassie brilliant blue [M: molecular mass maker; C: before induction (control group); 1: after 0.2 mM IPTG induction for 5 h at 37°C; 2: recombinant rDoenitin-1 purified by column, semi-permeable membrane dialysis and an ultrafiltration membrane].

**Figure 5:**
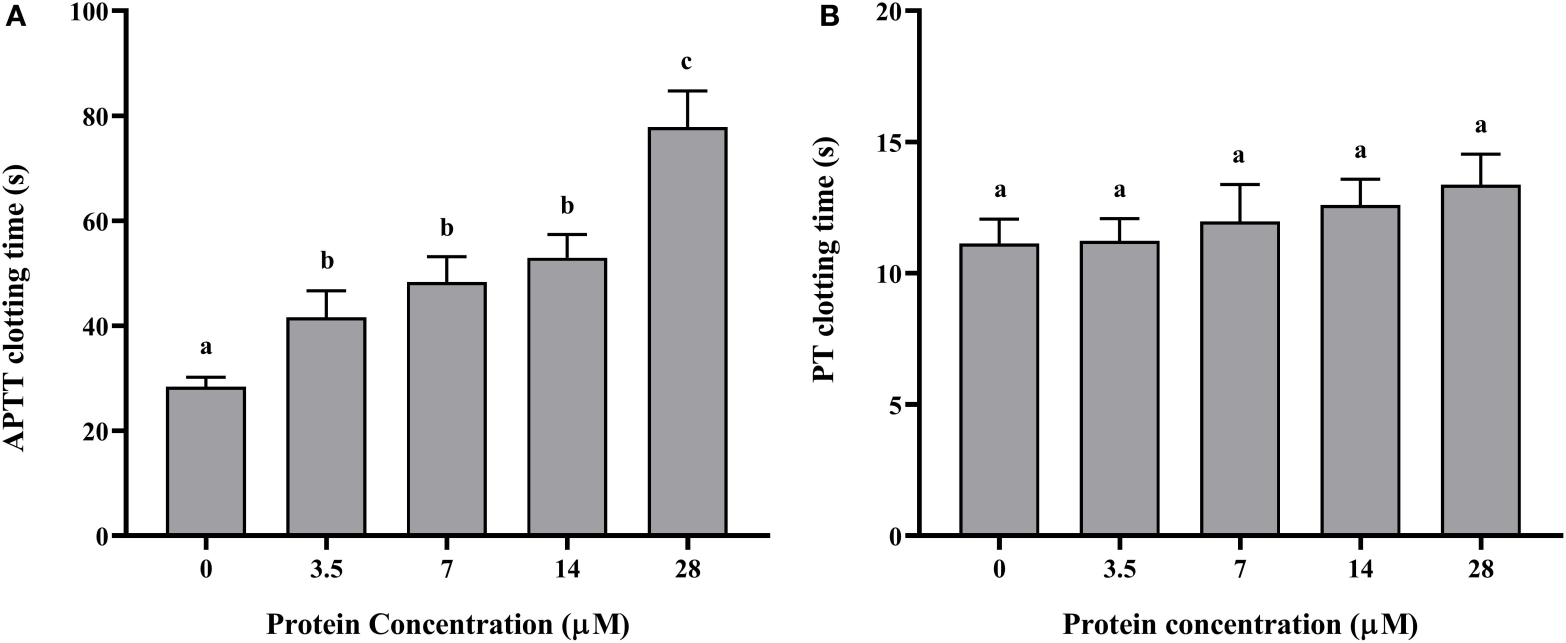
**(A)** APTT and **(B)** PT determination of rDoenitin-1. Coagulation time at different concentrations (0, 3.5, 7, 14, and 28 μM) of the recombinant protein. One-way ANOVA was conducted to analyze the data. Different letters indicate a significant difference between the two groups (*P* < 0.05).

The recombinant protein of *Doenitin-1* was successfully expressed through a prokaryotic expression system, and the molecular weight of the fusion protein was about 35 kDa (immature protein 15.8 kDa and label protein 19 kDa). The optimum elution concentration of imidazole for preliminary purification was 250 mM. After further purification by dialysis and concentration by ultrafiltration, the SDS-PAGE showed that purified rDoentitin-1 was free of impurities (Figure 4). The mass spectrometric data were searched in the UniProt protein database contain rDoenitin protein sequence using Proteome Discoverer 2.2 (Thermo Fisher Scientific). After enzymatic digestion, the peptides of the recombinant protein identified by LC-MS/MS correctly matched with the rDoenitin-1 protein (Table 2), which implied the *E. coli* prokaryotic expression system we constructed correctly expressed the rDoenitin protein of of the tick *H. doenitzi*. The normal APTT coagulation was 28.37 ± 1.47 s without rDoenitin-1. APTT coagulation was significantly prolonged after the addition of different concentrations of rDoenitin-1 (*P* < 0.05). When the concentration was 28 μM, APTT coagulation was extended to 77.8 ± 5.66 s (Figure 5A). However, PT coagulation was not significantly prolonged (Figure 5B). These results indicated that rDoenitin-1 can inhibit the intrinsic coagulation pathway but has no obvious effect on the extrinsic coagulation pathway.

**Table 2:** Mass spectrometry identification of rDoenitin-1.

**Sequence**	**Molecular weight (Da)**	**m/z**	**Theoretical *z***	**Score**
AFIPRYYFDVEK	1546.78	774.40	+2	14
GNENNFETLKECKDACTEPEK	2512.09	629.03	+4	12
YYFDVEKGQCEEFIYGGCK	2391.02	798.01	+3	10
ADFETGCKPAPETGHCLASHER	2460.04	616.02	+4	12

### Fibrinogen coagulation test

**Figure 6:**
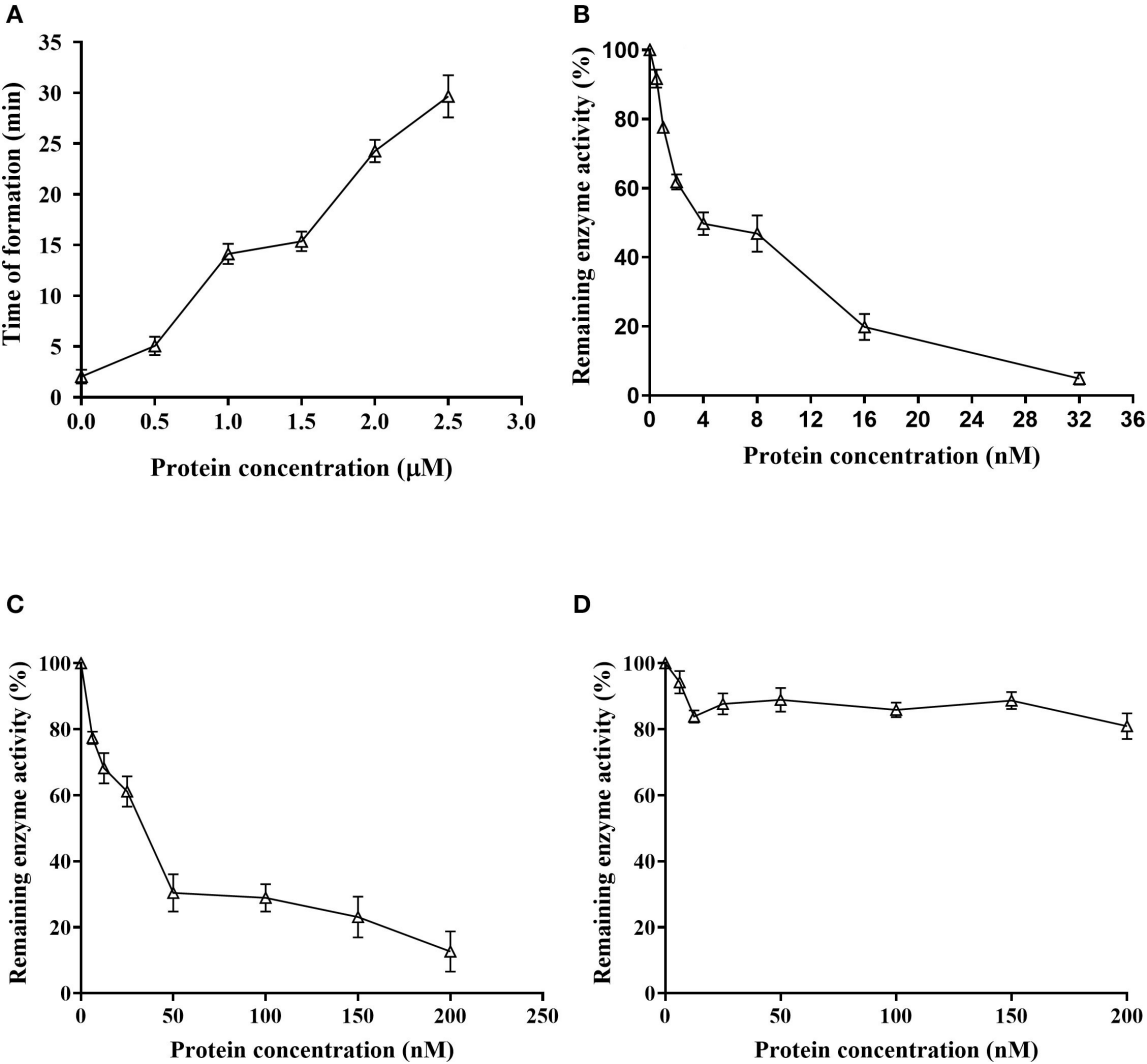
Function analysis of Doenitin-1. **(A)** rDoenitin-1 coagulation time at different concentrations (0, 0.5, 1, 1.5, 2, and 2.5 μM). Residual peptidase activity (%) of rDoenitin-1 on thrombin **(B)**, cathepsin G activity **(C)** and trypsin **(D)** by rDoenitin-1.

Fibrinogen coagulation time was 2.03 ± 0.68 min without rDoenitin-1, and fibrinogen coagulation occurred almost immediately. When the concentration of rDoenitin-1 was 2.5 μM, fibrinogen solidification time was extended to 29.67 ± 2.08 min (Figure 6A). The results showed that coagulation time was increased significantly with increase in rDoenitin-1 protein concentration, indicating that the inhibition of fibrinogen clot formation was gradually enhanced.

### Inhibitory effect of rDoenitin-1 on serine peptidase activity and its hemolytic activity

When rDoenitin-1 concentration was 0.5 nM, the residual peptidase activity of thrombin was 91.67%. When rDoenitin-1 concentration was 32 nM, the residual peptidase activity of thrombin was 4.85% (Figure 6B). The IC50 for thrombin was 6.546 nM (R^2^ = 0.9677). It can be seen that thrombin residual peptidase activity was gradually decreased with the increase in recombinant protein concentration, indicating inhibition of chromogenic substrate hydrolysis. When rDoenitin-1 concentration was 6.25 nM, the residual peptidase activity of cathepsin G was 77.39%. When rDoenitin-1 concentration was 200 nM, the residual peptidase activity of cathepsin G was 12.68 ± 5% (Figure 6C). The IC50 for cathepsin G was 30.01 nM (R^2^ = 0.9259). It showed that with increase in recombinant protein concentration, the residual peptidase activity of cathepsin G was gradually decreased, and no significant correlation was found between protein concentration and residual peptidase activity. There was no significant change in the residual peptidase activity of trypsin in the results (Figure 6D), indicating that rDoenitin-1 has no significant inhibitory effect on trypsin. The hemolytic rate of rDoenitin-1 was 4.25% (<5%), indicating that rDoenitin-1 had no significant hemolytic activity in healthy adult blood cells (Table 3).

**Table 3:** Hemolytic activity of rDoenitin-1.

**A_sample_**	**A_positive_**	**A_negative_**	**Hemolysis**	**Average**
0.214	2.756	0.116	3.712%	4.25%
0.246	2.772	0.126	4.535%	
0.240	2.750	0.122	4.490%	

### Versatile functions of *Doenitin-1* during feeding and reproduction of *H. doenitzi*

**Figure 7:**
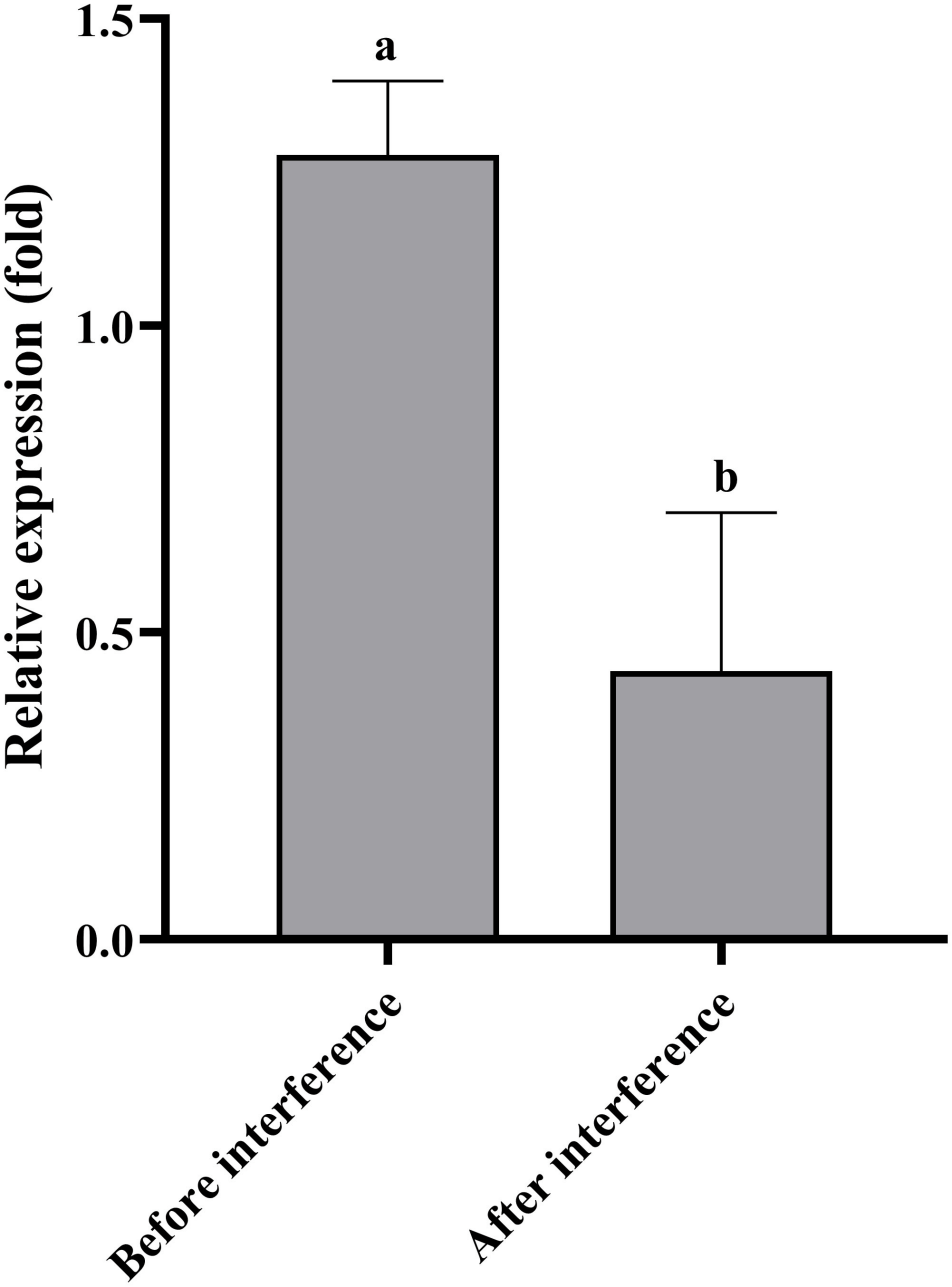
RNAi efficiency verification of *H. doenitzi*. Student's t test was conducted to analyze the data. Different letters indicate a significant difference between the two groups (*P* < 0.05).

The qPCR results showed that the relative expression level of *Doenitin-1* in the control group was 2.93 times higher than that of the interference group, indicating that the expression of *Doenitin-1* was reduced by 65.7% (Figure 7). The engorgement weight and engorgement rate of the female ticks were significantly reduced after *Doenitin-1* interference (*P* < 0.05). Compared with the GFP group, the egg hatchability was decreased significantly (*P* < 0.05), the egg hatchability of the GFP group was 85.36%, while it was only 46.67% in the experimental group. There were no significant changes in feeding time and 24-h attachment rate (*P* > 0.05) (Table 4). This result would suggest that *Doenitin-1* is not directly involved in the feeding site but may play a role in keeping the blood meal in liquid form in the gut.

**Table 4:** Effects of the RNAi of *Doenitin-1* on biological traits of *H. doenitzi*.

**Parameters**	**RNA interference**	
	**GFP**	***Doenitin-1***
No. test	60	60
24 h attachment rate (%)	36.67%	38.33%
Feeding time (d)	7.33	7.21
Engorgement weight (g)	0.2169 ± 0.0346	0.1632 ± 0.0836*
Engorgement rate (%)	74.14	43.24*
Egg hatchability (%)	85.36	46.67*

Note: one-way ANOVA was conducted to analyze the data; “^*^” indicates statistical differences (P < 0.05).

## Discussion

With the wide use of natural thrombin inhibitors, more and more thrombin inhibitors have been discovered from a great diversity of animals, including *Anopheles albimanus, Najahaje, Vespa bicolor* Fabricius, *Bombina microdeladigitora*, and *Schistosomiasis mansoni* (31–35). The wide use of thrombin inhibitors in different taxa suggests their importance in homeostasis regulation in most branches of life. To start blood feeding, ticks necessarily have to break their host's skin, which can cause a series of defense responses, including pain, hemostasis, complement activation, inflammation, and tissue repair of hosts (36, 37). In order to evade these defense mechanisms, ticks synthesize a range of anticoagulants, such as thrombin inhibitors (38). So far, a large number of anticoagulants have been identified that come from midguts, salivary glands, and hemolymph. These have a direct effect on blood-sucking to ensure a successful engorgement of ticks (23, 39). Besides, anticoagulants keep the blood liquid to enhance digestion by ticks. Currently, thrombin inhibitors have been found in some ticks, such as *R. microplus* (21), *H. longicornis* (9), and *Ornithodoros moubata* (16). Recent research shows that iripin-8 is a tick serpin with a conserved reactive center loop, which inhibits erythrocyte lysis to play a part in antihemostatic activity by complement. Moreover, it may mediate interference with host innate immunity (40).

In this study, a new anticoagulant was isolated from *H. doenitzi* and named *Doenitin-1*. It belongs to the Kunitz family and has two Kunitz domains, which are typical structures of thrombin inhibitors (41). The isoelectric point of *Doenitin-1* is close to that reported for the thrombin inhibitors hemalin (9) and boophilin (42), which bind to the negative charged exosite 1 of thrombin with their C-terminal domain to inhibit thrombin (21, 43). Kunitz peptidase inhibitors are widely distributed in animals and plants, and are involved in a series of different functions with their target peptidase inhibitors (41, 44). In this study, the inhibitory effect of recombinant protein on three serine proteases was tested. The IC50 of rhemathrin-1/2 isolated by Brahma et al. for thrombin was 46.13 and 40.05 μM, and that of Variegin was 0.99 nM (10, 13). The isolated simukunin from salivary glands of black flies has an IC50 of 217.4 nM for cathepsin G and 379.3 nM for trypsin (45). During the onset of tick feeding, the host produces immunologic rejection reaction and secretes a water-like fluid to the wound site to prevent the blood-sucking process of the tick (46). Studies found that active substances, such as thrombin inhibitors, secreted by salivary glands could not only cope with host immune defense and weaken the immunological rejection of ticks but also assist in the process of pathogen infection of the host (47). The rIris isolated from *I. ricinus* could significantly inhibit thrombin and cytokines, evading the host immune system (48). Currently, there are many studies on tick-derived peptidase inhibitors containing the Kunitz domain and not only have an antithrombin function but also can regulate host immunity (24, 25).

Anticoagulants play an important role in the blood-sucking process of blood-feeding arthropods. It can help ticks parasitize on the body of the host, smoothly sucking blood and finishing blood meal digestion. In this study, the engorgement body weight of female ticks injected with *Doenitin-1* dsRNA was significantly decreased, whereas the engorgement rate and egg hatchability were significantly decreased, which indicated that the decreased expression level of *Doenitin-1* could inhibit the blood-sucking of female ticks. During feeding, the rabbit ears inoculated with ticks in the experimental group were ulcerated and suppurated where inflammation was obvious, leading the death of the ticks and suggesting that *Doenitin-1* might inhibit host inflammation to some extent. It has been found that anticoagulant molecules in ticks could inhibit cytokines and affect the immune function of hosts (24, 48). After interference with hemalin, the blood-feeding period of ticks was prolonged and the engorgement rate was decreased, but the engorgement body weight, egg weight, and egg hatchability did not change significantly (9), while after interference with *Doenitin-1*, the engorgement body weight, engorgement rate, and egg hatchability were significantly reduced, indicating that the blood digestion and absorption processes were interfered. However, the 24-h attachment rate and feeding time showed no significant difference (*P* > 0.05) when compared with the control group. These results indicate that the thrombin inhibitor may regulate tick growth and development during blood-sucking. The hemolysis test showed that rDoenitin-1 had a hemolysis rate of 4.25%, which could be considered as having no hemolysis activity.

In this study, a novel anticoagulant from *H. doenitzi* named *Doenitin-1* was characterized. The BLAST result showed that it belongs to the Kunitz family and that it has two Kunitz domains. They are disulfide rich alpha+beta folds, which are typical structures of thrombin inhibitors (41). The C-terminal of doentin-1 bind to the negatively charged Exosite I site to inhibit thrombin, which are similar to Hemalin and Boophilin (9, 21, 42), which the C-terminal of thrombin inhibitors bind to the negatively charged Exosite I site to inhibit thrombin (21). In conclusion, the recombinant protein rDoenitin-1 has a significant inhibitory effect on specific peptidases. *Doenitin-1* plays an important role in oviposition, embryonic development, blood absorption, and digestion in adult ticks. The above results provide an important reference for development of new approaches for tick control and antithrombotic drugs. However, the limitation of this article is that the conformation of the natural protein and its enzymological characteristics have not been studied in-depth, so further study will be carried out using the natural protein of *Doenitin-1* isolated from ticks, which will elucidate the molecular basis of the enzymes and their key roles in regulation of tick development and the blood-sucking process.

## Data availability statement

The datasets presented in this study can be found in online repositories. The names of the repository/repositories and accession number(s) can be found in the article/supplementary material.

## Ethics statement

The animal study was reviewed and approved by Animal Ethics Committee of the Hebei Normal University.

## Author contributions

JZL, KW, and XY contributed to conception and design of the study. ZG and SZ organized the database. HL and YS performed the statistical analysis. JLL wrote the first draft of the manuscript. XS and ZY wrote sections of the manuscript. All the authors contributed to manuscript revision, read and approved the submitted version.

## Funding

This study was supported by the National Natural Science Foundation of China (31472050), Natural Science Foundation of Hebei Province (C2022205026), and Foundation of Hebei Educational Committee (ZD2020168, ZD2022008).

## Conflict of interest

The authors declare that the research was conducted in the absence of any commercial or financial relationships that could be construed as a potential conflict of interest.

## Publisher's note

All claims expressed in this article are solely those of the authors and do not necessarily represent those of their affiliated organizations, or those of the publisher, the editors and the reviewers. Any product that may be evaluated in this article, or claim that may be made by its manufacturer, is not guaranteed or endorsed by the publisher.
